# Stability of critical genetic lesions in human colorectal carcinoma xenografts.

**DOI:** 10.1038/bjc.1991.19

**Published:** 1991-01

**Authors:** H. A. McQueen, A. H. Wyllie, J. Piris, E. Foster, C. C. Bird

**Affiliations:** Department of Pathology, University Medical School, Edinburgh, UK.

## Abstract

**Images:**


Br. J. Cancer (1991), 63, 94-96 © Macmillan Press Ltd., 1991

### Stability of critical genetic lesions in human colorectal carcinoma xenografts

H.A. McQueen[1], A.H. Wyllie[1], J. Piris[1], E. Foster[2] & C.C. Bird[1]

[1]*Department of Pathology, University Medical School, Teviot Place, Edinburgh; and* [2]*MRC Human Genetics Unit, Crewe Road, Edinburgh, UK.*

Several genetic lesions are associated with the genesis of human colorectal carcinomas including mutational activation of Ki-*ras* and p53, and loss of heterozygosity involving presumptive oncosuppressor loci on 5q21, 17p13, and 18q22 (Fearon & Vogelstein, 1990). Abnormalities at the 17p13 locus are recorded in many types of tumours (Nigro *et al.*, 1989) but there is no evidence at present that the 5q and 18q loci are involved in sporadic neoplasia other than that of colorectal mucosa. Although the 17p13 locus is probably congenic with p53, and the product of the 18q22 gene appears to be a cell adhesion molecule, the function for all of these genes in oncosuppression is poorly understood. The ideal vehicle for assay and analysis of these unknown gene functions would be a colorectal cancer cell line in which the oncosuppressor genes were known to be aberrant or inactive. In theory, replacement of even one copy of the appropriate intact oncosuppressor gene might then restore phenotype to normality. Recent studies have demonstrated oncosuppression of this type on introduction of normal DNA from the retinoblastoma and Wilms' susceptibility loci, into tumour cell lines (Huang *et al.*, 1988; Weissman *et al.*, 1987). However, for none of the long-established colorectal cancer lines readily available is status at critical oncosuppressor loci known. Moreover long-established lines commonly acquire additional genetic rearrangements during culture (Brattain *et al.*, 1983; Park *et al.*, 1987), which may not be reversible by correction of the original oncosuppressor defect alone. We therefore set out to develop a series of lines from primary colorectal tumours, defined both in terms of status at oncosuppressor loci on chromosomes 5, 17 and 18, and the stability of each locus on serial passage.

Portions of carcinoma obtained from surgical resection specimens were xenografted as described by Lewko *et al.* (1989), into pairs of immunosuppressed (thymectomised, irradiated and arabinoside-C treated) CBA mice (Steel *et al.*, 1978), with the modification that portions no greater than 5 $mm^3$ were implanted dorsally at initial xenograft and at subsequent transplantations.

From a consecutive series of 28 cancers implanted, 11 grew to passage and eight were xenografted for more than five passages, one of which has now reached passage 16. At each serial passage excess tumour tissue was divided for analysis allowing the characterisation of xenografted tumours at various passage intervals, and the comparison of these with the primary tumour (portions of which were always stored both frozen and fixed at original resection). Haematoxylin and eosin stained paraffin sections of both primary tumour and xenografts fixed in 10% buffered formalin at all passages, showed that the original histological pattern for each primary tumour was conserved throughout serial passage. Although most of the lines fell into the class of adenocarcinomas of average degree of differentiation, extremes of both good and very poor differentiation were represented (Figure 1). No metastases were observed in mice.

To study the status of alleles closely linked to each of three putative colon-associated oncosuppressor loci, genomic DNA was prepared from frozen xenografted tumours at various passages, and compared with DNA from primary tumour tissue and normal colorectal mucosa or peripheral blood leukocytes from the donor patient. Methods for DNA preparation, Southern blotting and probes and conditions for hybridisation have been previously published (Ashton-

**Figure 1** Histological comparisons of original colorectal tumours with the corresponding xenografts. **a**, A poorly differentiated adenocarcinoma AGDU. **b**, Corresponding xenograft at 5th passage XAGDU4. **c**, A well differentiated adenocarcinoma MUCO. **d**, Corresponding xenograft at 11th passage XMUCO10. **e**, A moderately differentiated adenocarcinoma CHKE. **f**, Corresponding xenograft at 6th passage XCHKE5.

Correspondence: H.A. McQueen.
Received 29 May 1990; and in revised form 5 September 1990.

Rickardt *et al.*, 1989). Probes used were: for chromosome 5q: pYN5.48 (Nakamura *et al.*, 1988*a*), pEF5.44 (Dunlop *et al.*, 1990) π227 (Meera Khan *et al.*, 1988; Dunlop *et al.*, 1989), and two new probes pL5.62 and pL5.713 which map between π227 and YN5.48 (Y. Nakamura, personal communication); for 17p: pYNZ22 (Nakamura *et al.*, 1988*b*), pMCT35.1 (Carlson *et al.*, 1988); and for 18q: pBV15.65 (Vogelstein *et al.*, 1988). Almost all of these probes detect highly polymorphic sequences. pBV15.65 is situated within the 18q oncosuppressor gene, pMCT35.1 lies close to the p53 gene and the 5q probes are distributed through the APC locus over no more than 15mb.

Table I summarises allelic losses or retentions found for the parental normal and tumour DNA pairs at each locus. In all cases studied, the allelic status of xenograft DNA was unchanged from that of its primary tumour. For eight tumours this genetic stability was demonstrated over at least four xenograft passages (approximately 4 months) and in two (one of which showed allelic retention at 5q and 17p) over 11 passages (approximately 11 months). A variety of combinations of allelic losses and retentions are represented, broadly similar to those observed by ourselves and others in large unselected series of primary tumours (Ashton-Rickardt *et al.*, 1989; Vogelstein *et al.*, 1988). It appears from these observations that the selection pressures which encourage growth of human colorectal cancer xenografts in immunosuppressed animals do not include allelic losses around the oncosuppressor loci commonly affected in human carcinogenesis *in situ*.

Although our primary aim was to establish the stability of the lines at critical oncosuppressor loci, DNA ploidy was measured by flow cytometry as a global, if crude, index of total nuclear DNA content. Frozen tissues from both passage 5 xenografts and primary tumour were dissociated and stained with 0.62 M propidium iodide as described by Vindelov (1983), and analysed at an excitation wavelength of 488 nm in a Coulter Epics CS flow cytometer. On the basis of an internal chicken red blood cell standard, the presence of a human diploid peak could be confirmed in every case. This was given a ploidy value of 1, and any additional peaks were then assumed to be aneuploid, and their DNA index (DI) derived from their position relative to the diploid peak. In all cases, xenograft DI was similar to that of the relevant primary tumour (example shown in Figure 2), including four tumours remaining diploid after five or nine serial passages in mice, and one after 14 passages (15 months). A similarly high degree of conservation of genetic stability throughout xenograft passage in immunodeficient mice has been confirmed both karyotypically and at the genetic level by Lefrancois *et al.* (1989).

The demonstrated lack of genetic change for defined oncosuppressor loci qualifies these xenografts as favourable tumour models for analysis of oncosuppressor function, by re-introduction of the normal genes. Since growth *in vitro* is a prerequisite for genetic manipulation, tumour tissues removed immediately on to ice from animals at or following passage 5 were processed and seeded as primary cultures on collagen coated flasks with mouse feeder cells, as described by Paraskeva (1984). Epithelial cells from every xenograft line grew *in vitro* as flat islands of cells spreading radially across the collagen surface towards neighbouring islands (Figure 3). Two slightly faster growing lines were observed over three passages *in vitro*, and a third has now grown rapidly over 100 days, requiring a weekly split ratio of up to 1:20. On subcutaneous re-injection into immunosuppressed mice, these cells continued to show the same allelic status and morphology as the primary tumour.

In conclusion, the xenograft lines described above represent a variety of defined and stable combinations of oncosuppressor gene status and will therefore provide a useful resource for the study of the functions of the genes involved in human colorectal cancer, and their interactions with each other.

**Figure 2** Flow cytometric comparison of an aneuploid primary tumour (DNA index 1.7); arrowed, with the xenograft at passage 5 (DNA index 1.9). Diploid peak position is marked *.

**Figure 3** Phase contrast microscopy of XRHSP xenograft tumour epithelial cells growing in culture as islands (I) surrounded by feeder cells.

**Table I** Allelic status at three oncosuppressor loci, and DNA index for a set of 11 xenografts

| *Line* | *5q* | *17p* | *18q* | *DNA Index* |
|---|---|---|---|---|
| GRBO* | R (5) | R (4) | R (2) | 1.0 (5) |
| MUCO* | R (10) | R (10) | – | 1.0 (14) |
| RHSP* | L (10) | L (10) | – | 1.7–1.9 (12) |
| CHKE* | R (4) | R (4) | R (3) | 1.1–1.2 (9) |
| DABU* | R (5) | R (5) | NI | 1.0 (5) |
| ARNE* | R (4) | L (5) | L (5) | 1.5–1.6 (5) |
| MASM* | R (5) | R (5) | R (2) | 1.0 (9) |
| JOMcL* | R (4) | R (4) | NI | 1.0 (5) |
| JACA | L (2) | L (2) | L (2) | 1.6 |
| CARO | L (1) | L (1) | L (1) | 1.4 |
| JOWR | R (1) | R (1) | R (1) | 1.0 |

( ) = highest xenograft passage no. analysed; – = not done; NI = non-informative (patient homozygous for alleles studied); R = allele retained; L = allele lost; * = line xenografted over more than five passages. In all cases the allelic status of the xenografts throughout passage was identical with that of the primary tumour.

We thank Dr Y. Nakamura and Dr B. Vogelstein for their gifts of probes used in this study. This work was funded by grants from the Caledonian Research Foundation (formerly Inveresk Research Foundation), and the Cancer Research Campaign.

### References

ASHTON-RICKARDT, P.G., DUNLOP, M.G., NAKAMURA, Y. & 6 others (1989). High frequency of *apc* loss in sporadic colorectal carcinoma due to breaks clustered in 5q21–22. *Oncogene*, **4**, 1169.

BRATTAIN, M.G., MARKS, M.E., MCCOMBS, J., FINELY, W. & BRATTAIN, D.E. (1983). Characterization of human colon carcinoma cell lines isolated from a single primary tumour. *Br. J. Cancer*, **47**, 373.

CARLSON, M., NAKAMURA, Y., PAYSON, R. & 4 others (1988). Isolation and mapping of a polymorphic DNA sequence pMCT35.1 on chromosome 17p (D17S31). *Nucleic Acids Res.*, **16**, 783.

DUNLOP, M.G., STEEL, C.M., WYLLIE, A.H., BIRD, C.C. & EVANS, H.J. (1989). Linkage analysis in familial adenomatous polyposis: order of C11P11 (D5S71) and π227 (D5S37) loci at the apc gene. *Genomics*, **5**, 350.

DUNLOP, M.G., WYLLIE, A.H., NAKAMURA, Y., STEEL, C.M., EVANS, H.J. & BIRD, C.C. Genetic linkage map of 6 polymorphic DNA markers around the gene for familial adenomatous polyposis on chromosome 5. *Am. J. Human Genet.*, (in the press).

FEARON, E.R. & VOGELSTEIN, B. (1990). A genetic model for colorectal tumorigenesis. *Cell*, **61**, 759.

HUANG, H.S., YEE, J.Y., SHEW, J. & 5 others (1988). Suppression of the neoplastic phenotype by replacement of the RB gene in human cancer cells. *Science*, **242**, 1563.

LEFRANCOIS, D., OLSCHWANG, S., DELATTRE, O. & 4 others (1989). Preservation of chromosome and DNA characteristics of human colorectal adenocarcinomas after passage in nude mice. *Int. J. Cancer*, **44**, 871.

LEWKO, W.M., LADD, P., HUBBARD, D. & 7 others (1989). Tumour acquisition, propagation and preservation. The culture of human colorectal cancer. *Cancer*, **64**, 1600.

MEERA KHAN, P., TOPS, C.M.J., BROCK, M.V.D. & 10 others (1988). Close linkage of a highly polymorphic marker (D5S37) to familial adenomatous polyposis (FAP) and confirmation of FAP localization on chromosome 5q21–q22. *Hum. Genet.*, **79**, 183.

NAKAMURA, Y., LATHROP, M., LEPPERT, M. & 12 others (1988*a*). Localization of the genetic defect in familial adenomatous polyposis within a small region of chromosome 5. *Am. J. Human Genet.*, **43**, 638.

NAKAMURA, Y., BALLARD, L., LEPPERT, M. & 4 others (1988*b*). Isolation and mapping of a polymorphic DNA sequence (pYNZ22) on chromosome 17p (D17S30). *Nucleic Acids Res.*, **16**, 5707.

NIGRO, J.M., BAKER, S.J., PREISINGER, A.C. & 13 others (1989). Mutations in the p53 gene occur in diverse human tumour types. *Nature*, **342**, 705.

PARASKEVA, C., BUCKLE, B.G., SHEER, D. & WIGLEY, C. (1984). The isolation and characterization of colorectal epithelial cell lines at different stages in malignant transformation from familial polyposis coli patients. *Int. J. Cancer*, **34**, 49.

PARK, J., OIE, H.K., SUGARBAKER, P.H. & 4 others (1987). Characteristics of cell lines established from human colorectal carcinoma. *Cancer Res.*, **47**, 6710.

STEEL, G.G., COURTENAY, V.D. & ROSTOM, A.Y. (1978). Improved immune-suppression techniques for the xenografting of human tumours. *Br. J. Cancer*, **37**, 224.

VINDELOV, L.L., CHRISTENSEN, I.J. & NISSEN, N.I. (1983). A detergent trypsin method for the preparation of nuclei for flow cytometric DNA analysis. *Cytometry*, **3**, 323.

VOGELSTEIN, B., FEARON, E.R., STANLEY, B.A. & 8 others (1988). Genetic alterations during colorectal tumour development. *New Engl. J. Med.*, **319**, 525.

WEISSMAN, B.E., SAXON, P.J., PASQUALE, S.R., JONES, G.R., GEISER, A.G. & STANBRIDGE, E.J. (1987). Introduction of a normal human chromosome 11 into a Wilms' tumour cell line controls its tumorigenic expression. *Science*, **236**, 175.


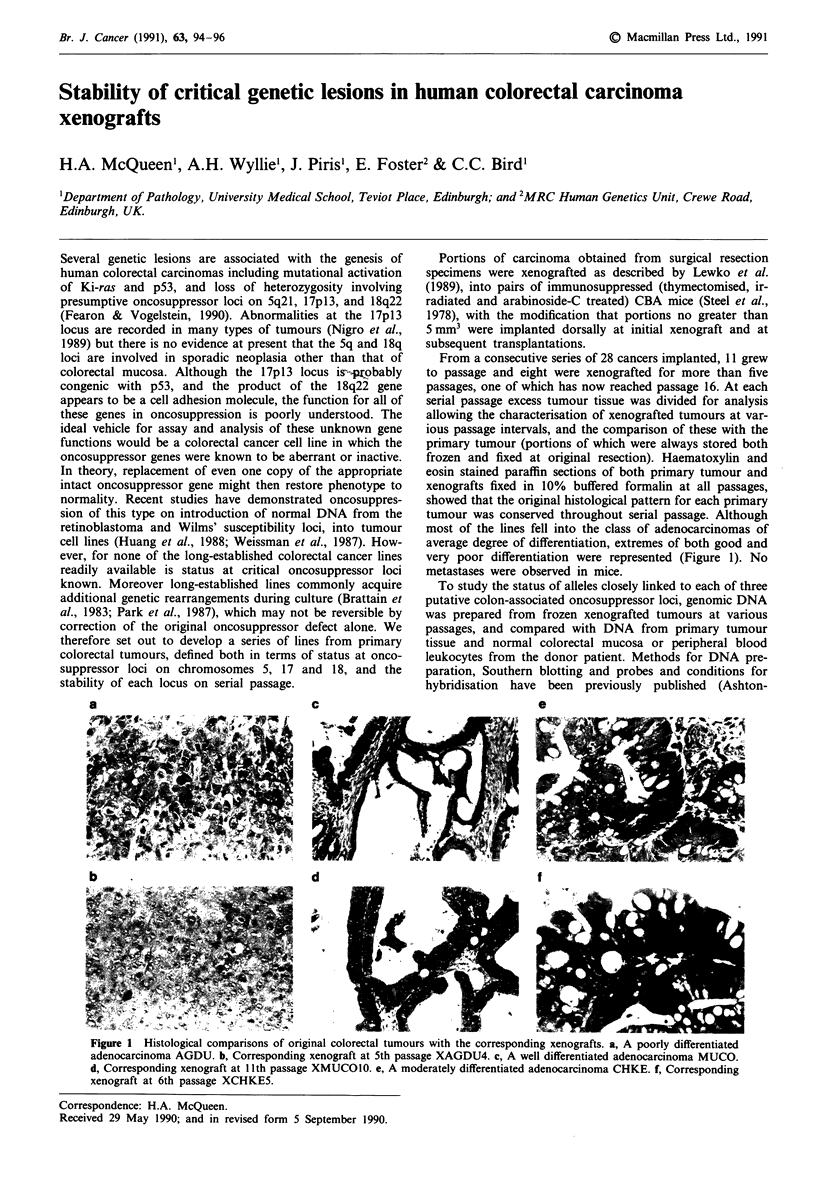

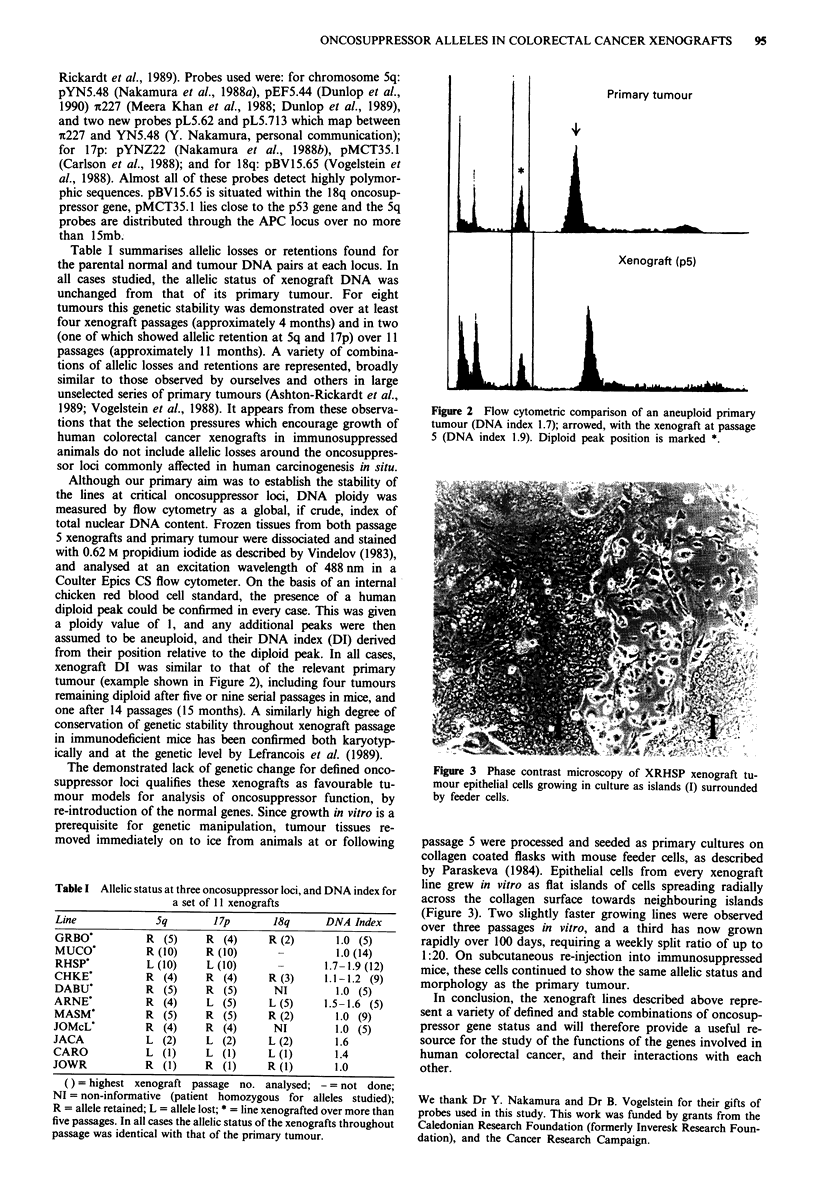

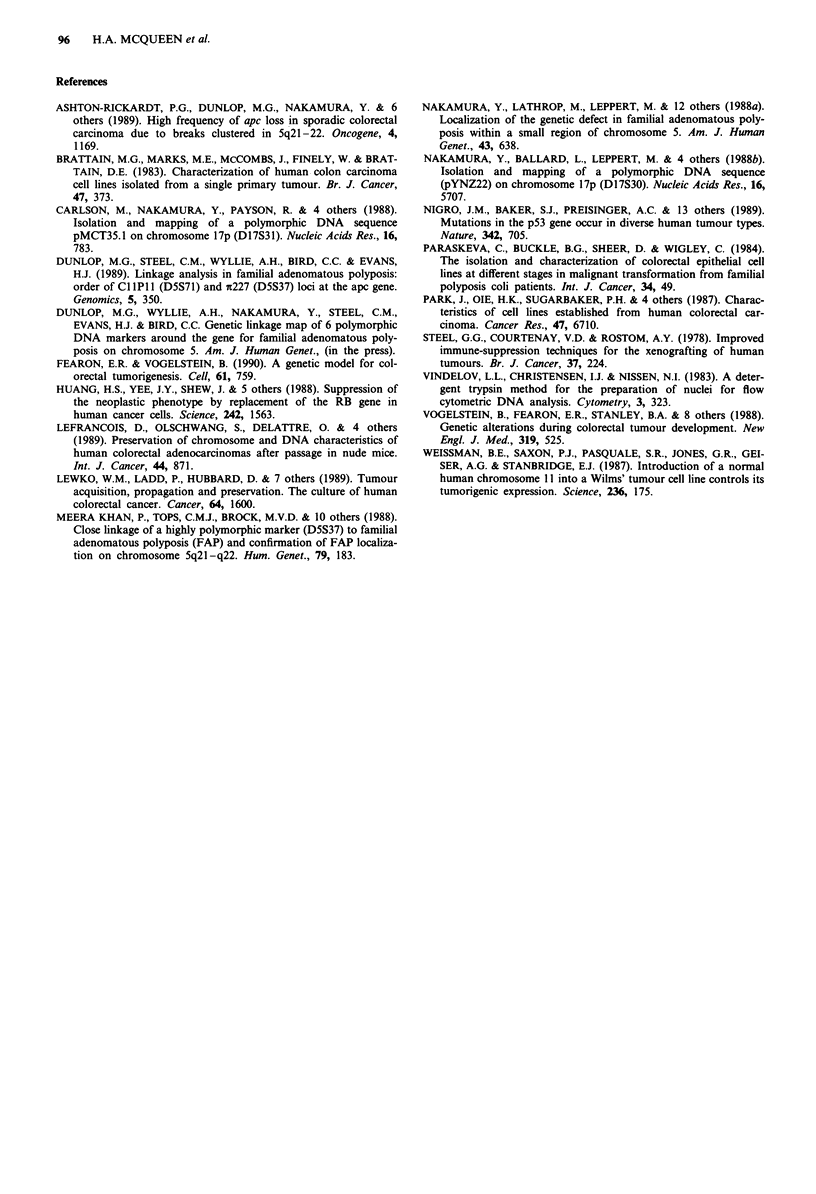


## References

[OCR_00238] Ashton-Rickardt P. G., Dunlop M. G., Nakamura Y., Morris R. G., Purdie C. A., Steel C. M., Evans H. J., Bird C. C., Wyllie A. H. (1989). High frequency of APC loss in sporadic colorectal carcinoma due to breaks clustered in 5q21-22.. Oncogene.

[OCR_00246] Brattain M. G., Marks M. E., McCombs J., Finely W., Brattain D. E. (1983). Characterization of human colon carcinoma cell lines isolated from a single primary tumour.. Br J Cancer.

[OCR_00250] Carlson M., Nakamura Y., Payson R., O'Connell P., Leppert M., Lathrop G. M., Lalouel J. M., White R. (1988). Isolation and mapping of a polymorphic DNA sequence pMCT35.1 on chromosome 17p [D17S31].. Nucleic Acids Res.

[OCR_00256] Dunlop M. G., Steel C. M., Wyllie A. H., Bird C. C., Evans H. J. (1989). Linkage analysis in familial adenomatous polyposis: order of C11P11 (D5S71) and pi 227 (D5S37) loci at the apc gene.. Genomics.

[OCR_00267] Fearon E. R., Vogelstein B. (1990). A genetic model for colorectal tumorigenesis.. Cell.

[OCR_00271] Huang H. J., Yee J. K., Shew J. Y., Chen P. L., Bookstein R., Friedmann T., Lee E. Y., Lee W. H. (1988). Suppression of the neoplastic phenotype by replacement of the RB gene in human cancer cells.. Science.

[OCR_00278] Lefrançois D., Olschwang S., Delattre O., Muleris M., Dutrillaux A. M., Thomas G., Dutrillaux B. (1989). Preservation of chromosome and DNA characteristics of human colorectal adenocarcinomas after passage in nude mice.. Int J Cancer.

[OCR_00284] Lewko W. M., Ladd P., Hubbard D., He Y. J., Vaghmar R., Husseini S., Chang L., Moore M., Thurman G. B., Oldham R. K. (1989). Tumor acquisition, propagation, and preservation. The culture of human colorectal cancer.. Cancer.

[OCR_00289] Meera Khan P., Tops C. M., vd Broek M., Breukel C., Wijnen J. T., Oldenburg M., vd Bos J., van Leeuwen-Cornelisse I. S., Vasen H. F., Griffioen G. (1988). Close linkage of a highly polymorphic marker (D5S37) to familial adenomatous polyposis (FAP) and confirmation of FAP localization on chromosome 5q21-q22.. Hum Genet.

[OCR_00299] Nakamura Y., Ballard L., Leppert M., O'Connell P., Lathrop G. M., Lalouel J. M., White R. (1988). Isolation and mapping of a polymorphic DNA sequence (pYNZ22) on chromosome 17p [D17S30].. Nucleic Acids Res.

[OCR_00295] Nakamura Y., Lathrop M., Leppert M., Dobbs M., Wasmuth J., Wolff E., Carlson M., Fujimoto E., Krapcho K., Sears T. (1988). Localization of the genetic defect in familial adenomatous polyposis within a small region of chromosome 5.. Am J Hum Genet.

[OCR_00305] Nigro J. M., Baker S. J., Preisinger A. C., Jessup J. M., Hostetter R., Cleary K., Bigner S. H., Davidson N., Baylin S., Devilee P. (1989). Mutations in the p53 gene occur in diverse human tumour types.. Nature.

[OCR_00310] Paraskeva C., Buckle B. G., Sheer D., Wigley C. B. (1984). The isolation and characterization of colorectal epithelial cell lines at different stages in malignant transformation from familial polyposis coli patients.. Int J Cancer.

[OCR_00316] Park J. G., Oie H. K., Sugarbaker P. H., Henslee J. G., Chen T. R., Johnson B. E., Gazdar A. (1987). Characteristics of cell lines established from human colorectal carcinoma.. Cancer Res.

[OCR_00321] Steel G. G., Courtenay V. D., Rostom A. Y. (1978). Improved immune-suppression techniques for the exongrafting of human tumours.. Br J Cancer.

[OCR_00326] Vindeløv L. L., Christensen I. J., Nissen N. I. (1983). A detergent-trypsin method for the preparation of nuclei for flow cytometric DNA analysis.. Cytometry.

[OCR_00331] Vogelstein B., Fearon E. R., Hamilton S. R., Kern S. E., Preisinger A. C., Leppert M., Nakamura Y., White R., Smits A. M., Bos J. L. (1988). Genetic alterations during colorectal-tumor development.. N Engl J Med.

[OCR_00338] Weissman B. E., Saxon P. J., Pasquale S. R., Jones G. R., Geiser A. G., Stanbridge E. J. (1987). Introduction of a normal human chromosome 11 into a Wilms' tumor cell line controls its tumorigenic expression.. Science.

